# Anlotinib combined with whole-brain radiotherapy in non-small cell lung cancer with multiple brain metastases that progressed or developed after at least one lines of prior treatment

**DOI:** 10.3389/fonc.2023.1169333

**Published:** 2023-09-12

**Authors:** Cheng Kong, Shaorong Yu, Pudong Qian, Xue Song, Jing Wen, Ming Jiang, Jun Zhu, Jianhua Xu, Lijun Zhao, Zhen Guo, Jianfeng Wu, Xia He, Xiangzhi Zhu

**Affiliations:** ^1^ Department of Radiation Oncology, Jiangsu Cancer Hospital and Nanjing Medical University Affiliated Cancer Hospital and Jiangsu Institute of Cancer Research, Nanjing, China; ^2^ Department of Medical Oncology, Jiangsu Cancer Hospital and Nanjing Medical University Affiliated Cancer Hospital and Jiangsu Institute of Cancer Research, Nanjing, China; ^3^ Department of Radiology, Jiangsu Cancer Hospital and Nanjing Medical University Affiliated Cancer Hospital and Jiangsu Institute of Cancer Research, Nanjing, China

**Keywords:** non-small cell lung cancer, brain metastases, later-line, whole-brain radiotherapy, antiangiogenic TKIs

## Abstract

**Background:**

Intracranial metastasis that failed standard systematic treatment is common in advanced non-small cell lung cancer (NSCLC), contributing significantly to morbidity and mortality. The aim of this study was to evaluate the efficacy and safety of anlotinib combined with whole-brain radiotherapy (WBRT) for NSCLC with brain metastases (BMs) that progressed or developed after at least one line of prior treatment and compare the outcomes with that of the contemporary institutional control.

**Methods:**

NSCLC patients with multiple BMs that progressed or developed after at least one line of prior systematic treatment and treated with WBRT subsequently between 2019 and 2021 were selected retrospectively for analysis. Based on whether concurrent anlotinib had been used in combination with WBRT, the cases were divided into the anlotinib group and control group. The primary endpoints were intracranial progression-free survival (iPFS) and safety.

**Results:**

A total of 76 patients met the inclusion criteria of the study. Of the 76 patients, 34 received concurrent WBRT and anlotinib followed by anlotinib maintenance and 42 were treated with WBRT alone or in combination with other systemic agents at the physicians’ discretion. The median follow-up for the entire cohort was 21 months. The median iPFS for the anlotinib and control group was 6.7 months (95% CI, 4.6–9.9) and 5.3 months (95% CI, 4.0–6.5), respectively (log-rank *P* = 0.04). There was no difference in overall survival between the two groups (log-rank *P* = 0.38). In the anlotinib group, treatment-related adverse events were reported in 15 patients (44.1%), with acute or late grade 3–5 adverse events identified in 14.7% of patients (n = 5).

**Conclusions:**

WBRT plus anlotinib, as a convenient chemo-free regimen, may represent an overall safe and effective procedure in advanced NSCLC with multiple BMs that progressed or developed after standard systematic treatment.

## Introduction

As many as 40% of patients diagnosed with non-small cell lung cancer (NSCLC) will develop brain metastases (BMs) during the course of their disease, and this risk may be even greater along with prolonged survival from evolved systemic agents that can achieve better extracranial control and the increased quality of central nervous system imaging (1–4). Intracranial metastasis that failed standard systematic treatment is also common in advanced NSCLC, contributing significantly to morbidity and mortality.

In addition to systemic therapies, the historically developed treatment of whole-brain radiotherapy (WBRT) continues to be a very widely used option in the management of patients with multiple BMs, particularly for whom surgery or stereotactic radiosurgery (SRS) is not recommended. However, there might be no clear benefit from WBRT compared with supportive care alone, given the poor overall survival (OS) outcomes typically expected in such WBRT-indicated patients with innumerable BMs, low Radiation Therapy Oncology Group (RTOG) disease-specific Graded Prognostic Assessment scores, or medical contraindications. In the QUARTZ trial (5), the sole phase 3 randomized controlled trial addressing the effcacy of WBRT compared with optimal supportive care alone in patients with brain-metastatic NSCLC who were not candidates for SRS, no significant differences in OS and overall quality of life were observed between the two groups. Innovative treatment strategies are urgently needed, since the therapeutic efficacy in patients with brain-metastatic NSCLC who are likely to have poor prognosis remains unsatisfactory when treated with WBRT alone.

Neoangiogenesis is crucial for BM growth, particularly in circumstances where they appear to progress rapidly. The combination of WBRT and antiangiogenesis inhibitors may represent a potentially beneficial strategy in patients typically either without actionable genomic alterations or resistant to prior classic targeted agents and having symptomatic multiple BMs. The REBECA phase I study has demonstrated the feasibility of combined standard WBRT and bevacizumab and provided preliminary efficacy data in patients with unresectable BMs from solid tumors (6).

Anlotinib is a novel orally administered antiangiogenesis inhibitor, which functions by inhibiting the vascular endothelial growth factor (VEGF) receptors (1/2/3) and other major tyrosine kinase receptors, such as FGFR1-4, PDGFR a/b, c-Kit, and FLT3 (7). *Post-hoc* analysis of a phase 3 randomized control trial (ALTER0303) has demonstrated that anlotinib can benefit patients with brain-metastatic NSCLC who failed at least second-line therapy and is highly potent in the management of intracranial lesions (8). Based on these findings, WBRT combined with anlotinib may be a good alternative option for treating BMs from NSCLC and deserves further investigation. Since 2019, we began to treat brain-metastatic patients with this strategy at our institution. In this study, the efficacy and safety of the combination of WBRT plus anlotinib for brain-metastatic NSCLC in the second-or-more-line setting were evaluated, and the survival outcomes were compared with those of contemporary institutional control.

## Materials and methods

### Design and patients

This was a retrospective study for patients with histologically proven NSCLC with BMs confirmed by MRI. The study protocol was reviewed and approved by the institutional review board and ethics committee at Jiangsu Cancer Hospital.

An institutional database was queried to identify eligible patients. Eligible patients had the following inclusion criteria: 1) aged 18 years or older; 2) presenting with locally advanced or metastatic NSCLC originally; 3) multiple BMs that progressed or developed after at least one line of prior systematic treatment (chemotherapy/targeted therapy/immunotherapy) and treated with WBRT subsequently between April 2019 and Jan 2021; and 4) either without sensitizing Epidermal Growth Factor Receptor (EGFR)/anaplastic lymphoma kinase (ALK)/ROS proto-oncogene 1 (ROS1) genomic aberration or resistant to prior classic targeted agents and no other active molecular-targeted drugs. Patients with 10 or fewer BMs where numerically SRS may be a treatment option were included based on the clinicians’ judgment on the global health status of patients. Pragmatically and inclusively, patients with a Karnofsky Performance Status (KPS) of <70 were also eligible. The main exclusion criteria were the following: 1) up-front RT to the brain; 2) previous use of antiangiogenic tyrosine kinase inhibitors (TKIs), such as apatinib and anlotinib (but previous treatment with bevacizumab was permitted); 3) synchronous meningeal carcinomatosis and/or spinal canal metastasis; 4) previous malignancy within 3 years before this study (other than *in situ* cancers or non-melanomatous skin cancers); 5) manifestation of slow intracranial progression with adequately controlled extracranial disease after up-front effective targeted therapy in patients with activating genomic aberrations; and 6) with active hemorrhage or at the risk of hemorrhage.

According to whether or not concurrent anlotinib had been used in combination with WBRT, eligible cases were divided into the anlotinib group (an-WBRT) and control group (con-WBRT). The control group received WBRT alone or combined with systemic agents, other than antiangiogenic small-molecule TKIs such as anlotinib, at the discretion of treating physicians. Notably, some of the patients in the an-WBRT group were derived from a prospective collection of a single-arm phase 2 trial (chictr.org.cn identifier: ChiCTR1900022093) at our institution, the results of which will be published separately in the future.

### Treatment in the anlotinib group

Patients were treated with concurrent anlotinib and WBRT followed by anlotinib consolidation at intracranial progression per protocol. Anlotinib (12 mg recommended, 10 mg or 8 mg acceptable if clinically indicated; Chia Tai Tianqing Pharmaceutical Group Co., Ltd.) was orally administered once daily on days 1–14 per 3-week cycle. Treatment continued until disease progression, intolerance, or patients’ withdrawal of consent. WBRT was defined as 30 Gy in 10–12 daily fractions ideally given over 2–2.5 weeks with a 6-MV linear accelerator with two parallel opposed fields. Subsequent local boost doses in 2–5 fractions were permitted at the clinician’s discretion. Hippocampal avoidance RT technique was not used.

### Assessment

Brain MRIs were used to evaluate the intracranial disease at baseline and during follow-up period every 2–3 months. The intracranial response was assessed per Response Evaluation Criteria in Solid Tumors 1.1. In order to improve the reliability of the result, radiologists responsible for the assessment were blinded to the patient’s treatment assignment when evaluating intracranial progression independently. More rigid application of imaging criteria was recommended for the determination of progressive disease (PD). Patients with radiologic evidence of PD could continue anlotinib in combination with other agents or not provided that they would benefit from continuous treatment. Adverse events (AEs) were graded according to the US National Cancer Institute’s Common Terminology Criteria for Adverse Events (version 4.0). Acute AEs were defined as those arising within 3 months of onset of anlotinib oral administration while late AEs after 3 months.

### Endpoints

The primary endpoints were intracranial progression-free survival (iPFS) and safety. The secondary endpoint was OS. iPFS was calculated from the date of intracranial progression diagnosis before WBRT until the date of growth of a previous lesion, the development of a new lesion, or death from any cause. OS was calculated from the date of intracranial progression diagnosis before WBRT until the date of death.

### Statistics

Characteristics of patients in the two groups were compared descriptively and with the χ^2^ test for categorical variables and analysis of variance for continuous variables. Kaplan–Meier analysis was used to estimate OS and iPFS, whereas log-rank testing was used to assess for differences. Both univariate and multivariate Cox proportional hazards analyses were performed to identify predictors for OS and iPFS separately. All of the significant predictors of either iPFS or OS in univariate models and the variable of interest (treatment assignment) were assessed by corresponding multivariate Cox proportional hazard analysis. The following prognostic factors were evaluated: stage at diagnosis (IV vs. I–III), smoking status (current/former vs. never), histology (other vs. adenocarcinoma), sex (male vs. female), age (continuous), mutation type (EGFR 19/21 or ALK mutation vs. wild type or unknown), treatment assignment (an-WBRT vs. con-WBRT), line number of assigned treatment (third line or later vs. second line), KPS (60 or less vs. 70 or more), BM status (newly diagnosed vs. PD), number of BMs (>10 vs. 2–10), size of largest BM (continuous), cyst-like BMs (No vs. Yes), peritumoral edema from BMs (moderate or severe vs. absent or mild), extracranial metastases at time of intracranial progression (Yes vs. No), number of extracranial metastatic organs (3–7 vs. 0–2), number of extracranial lesions (5 or more vs. 0–4), liver metastases (no vs. yes), primary NSCLC status (uncontrolled vs. controlled or absent), local boost at WBRT (Yes vs. No), dexamethasone equivalent dose used over WBRT (continuous), and immune checkpoint inhibitor (ICI) combined with assigned treatment (yes vs. no). Statistical analyses were performed using STATA 16 (Stata, College Station, TX, USA).

## Results

**Figure 1:**
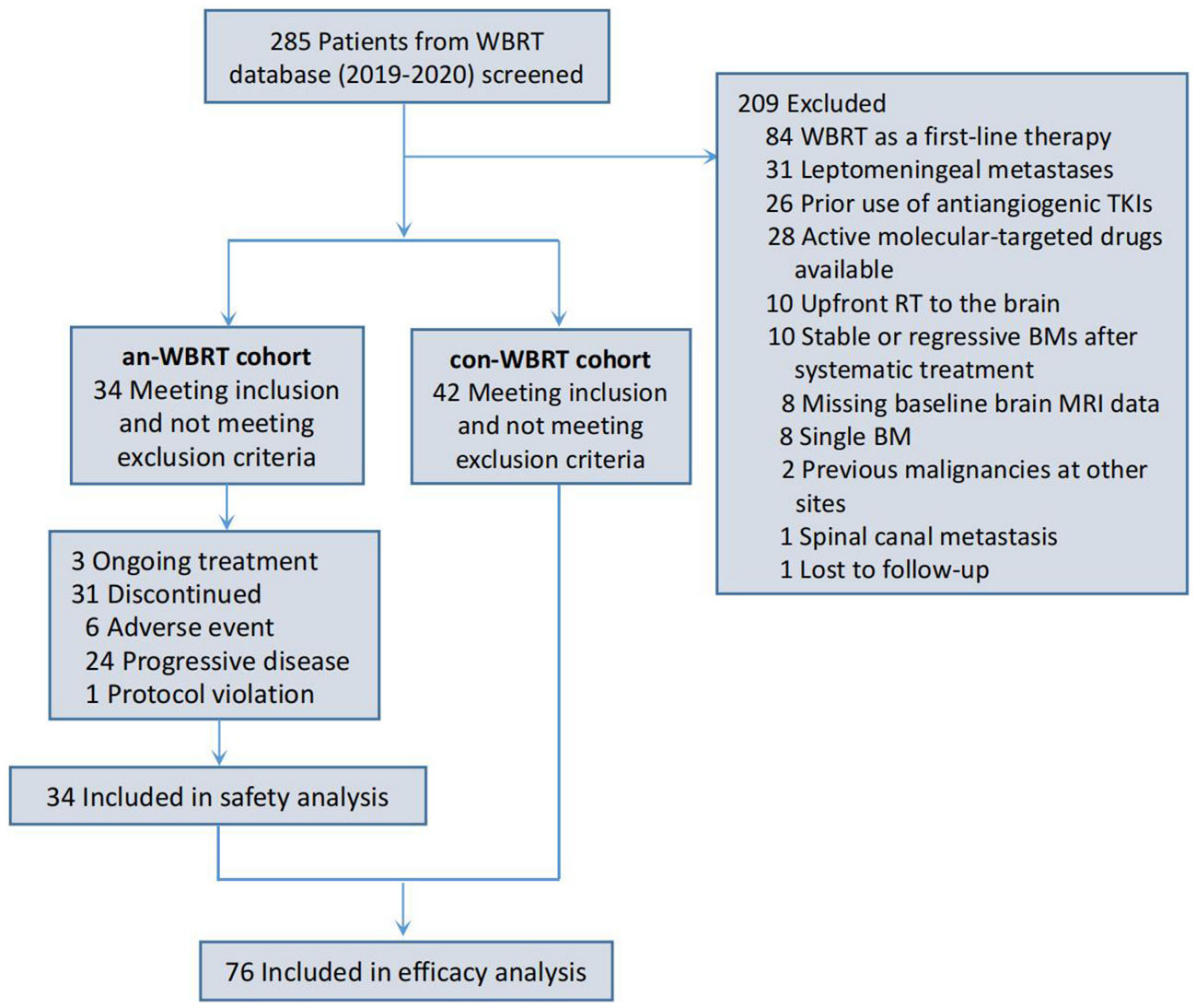
Patient flowchart.

A total of 76 patients were identified (**Figure 1**). Of them, 34 received concurrent WBRT and anlotinib followed by anlotinib maintenance, and 42 were treated with WBRT alone or combined with other systemic agents at the discretion of physicians. The final follow-up date was 30 January 2022. The median follow-up time for the entire cohort of 76 patients was 21 months (interquartile range, 14–22). Patient characteristics are listed in **Table 1**. The baseline characteristics of the two groups were generally well balanced (**Table 1**). The different corticosteroid doses for the management of symptoms over WBRT were observed. Patients in the con-WBRT group were more likely to receive >69 mg dexamethasone equivalent dose (60% con-WBRT vs. 38% an-WBRT), albeit with a marginal level of significance (*P* = 0.065).

**Table 1:** Patient characteristics.

	con-WBRT (n=42)		an-WBRT (n=34)		
Characteristic	No.	%	No.	%	*P*
Age at WBRT, years					
Median	60.5		61		0.815
< 61	21	50	16	47	0.799
≥ 61	21	50	18	53	
Sex					
Female	22	52	16	47	0.645
Male	20	48	18	53	
Stage at diagnosis					
I–III	11	26	11	32	0.556
IV	31	74	23	68	
KPS					
≤ 60	13	31	11	32	0.896
≥ 70	29	69	23	68	
Smoking status					
Never	24	57	18	53	0.714
Current/former	18	43	16	47	
Histology					
Adenocarcinoma	37	88	29	85	0.719
Others	5	12	5	15	
EGFR 19/21 or ALK mutation					
Yes	20	48	11	32	0.178
Wild type or unknown	22	52	23	68	
Line number of assigned treatment					
Second	18	43	12	35	0.502
Third or later	24	57	22	65	
BM status					
Progressive disease	22	52	16	47	0.645
Newly diagnosed	20	48	18	53	
Number of BMs					
2–10	23	55	17	50	0.679
>10	19	45	17	50	
Size of largest BM					
≤ 1.7 cm	21	50	13	38	0.305
> 1.7 cm	21	50	21	62	
BMs with cyst formation					
Yes	30	71	21	62	0.373
No	12	29	13	38	
Peritumoral edema from BMs					
Absent or mild	19	45	16	47	0.874
Moderate or severe	23	55	18	53	
Extracranial metastases at time of intracranial progression					
No	10	24	7	21	0.738
Yes	32	76	27	79	
Number of extracranial metastatic organs					
0–2	23	55	22	65	0.38
3–7	19	45	12	35	
Number of extracranial lesions					
0–4	14	33	11	32	0.928
≥ 5	28	67	23	68	
Liver metastases					
Yes	10	24	5	15	0.321
No	32	76	29	85	
Primary NSCLC status					
Controlled or absent	23	55	21	62	0.539
Uncontrolled	19	45	13	38	
Local boost at WBRT					
No	31	74	27	79	0.568
Yes	11	26	7	21	
Dexamethasone equivalent dose over WBRT					
≤ 69 mg	17	40	21	62	0.065
> 69 mg	25	60	13	38	
ICI combined with assigned treatment					
Yes	7	17	8	24	0.455
No	35	83	26	76	

WBRT, whole-brain radiotherapy; KPS, Karnofsky Performance Status; brain metastases, BMs; NSCLC, non-small cell lung cancer; ICI, immune checkpoint inhibitor.

As the use of other systemic therapies combined with WBRT and anlotinib was not strictly limited, eight patients also received additional immunotherapy and four received chemotherapy in the an-WBRT group (**Supplementary** **Table S1**). Similarly, the types of systemic therapies used in combination with WBRT in the con-WBRT group were shown in **Supplementary** **Table S2**. Of note, 11 patients from the con-WBRT group crossed over to receive anlotinib at the time of progression afterward.

### Intracranial progression

An event of intracranial disease progression or death from any cause had occurred in 68 patients (89.5%) in the entire cohort at the time of data cutoff. Of them, the event was determined with the radiological tumor assessment documenting the presence of PD in 23 (30.3%) and with clinical assessment in one (1.3%). The latter one presenting with severe headache that resolved following subsequent intrathecal chemotherapy was presumed to have a PD of leptomeningeal metastasis, although characteristic abnormal MRI findings were not detected. Death was noted as an event of iPFS in 44 patients (57.9%) for whom no prior unambiguous evidence of intracranial PD was available. Death and radiologic evidence recorded as an event of iPFS correspond to 23 cases and 16 cases in the con-WBRT group, as well as 21 and 7 in the an-WBRT group, respectively (**Supplementary** **Table S3**).

To deal with potential ascertainment bias that may be magnified toward the end of life and thus cause misleading interpretation of primary outcome, for the subset using the death time as an iPFS event, time intervals from the last radiologic assessment of brain performed after completing WBRT, or if lack from the end of WBRT, to late-death were compared between the two groups (**Supplementary Tables S4, S5**). We found that both types of intervals were generally symmetrical across treatment arms.

**Figure 2:**
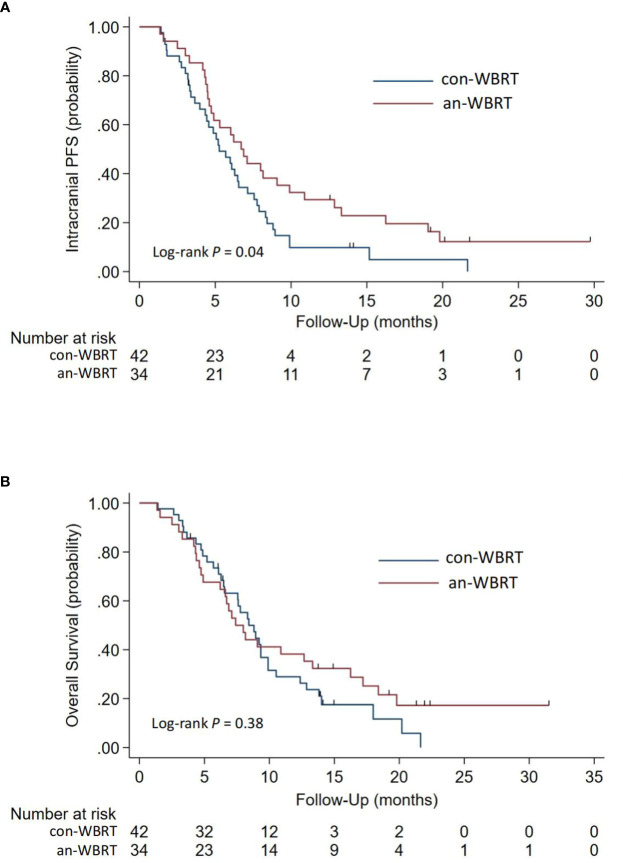
Kaplan-Meier survival curves for **(A)** Intracranial Progression-free Survival and **(B)** Overall survival.

The median time to intracranial progression or death from any cause for the con-WBRT and an-WBRT groups was 5.3 months (95% CI, 4.0–6.5) and 6.7 months (95% CI, 4.6–9.9), respectively (log-rank *P* = 0.04, **Figure 2A**). The results of the univariate and multivariate Cox regression survival analyses are shown in **Table 2**. In the multivariate Cox model, merely treatment assignment was associated with trends toward lower probability of intracranial progression after controlling for the mutation type, line number of assigned treatment, and KPS, with an adjusted hazard ratio (HR) of 0.57 (95% CI, 0.33–0.99; *P* = 0.044).

**Table 2:** Univariable and multivariable analyses of covariables associated with iPFS.

	Univariable Analysis			Multivariable Analysis		
Variable	HR	95% CI	*P*	HR	95% CI	*P*
an-WBRT vs. con-WBRT	0.60	0.37–0.98	0.043	0.57	0.33–0.99	0.044
Age	0.99	0.97–1.01	0.364			
Sex						
Male vs. female	0.67	0.41–1.09	0.105			
Histology						
Other vs. adenocarcinoma	0.96	0.44–2.10	0.909			
Smoking status						
Current/former vs. never	0.79	0.48–1.28	0.335			
Stage at diagnosis						
IV vs. I–III	1.16	0.68–1.97	0.595			
Mutation type						
EGFR 19/21 or ALK mutation vs. wild type or unknown	1.81	1.09–3.01	0.021	1.28	0.73–2.24	0.389
Line number of assigned treatment						
Third or later vs. second	1.68	1.02–2.79	0.043	1.70	0.98–2.94	0.058
KPS						
≤ 60 vs. ≥ 70	1.76	1.04–2.99	0.036	1.63	0.95–2.78	0.076
BM status						
Newly diagnosed vs. progressive disease	0.85	0.53–1.39	0.523			
Number of BMs						
> 10 vs. 2–10	1.23	0.76–1.99	0.390			
Size of largest BM	0.84	0.66–1.07	0.157			
BMs with cyst formation						
No vs. Yes	1.24	0.75–2.07	0.402			
Peritumoral edema from BMs						
Moderate or severe vs. absent or mild	0.71	0.44–1.15	0.164			
Extracranial metastases at time of intracranial progression						
Yes vs. No	1.27	0.71–2.26	0.419			
Number of extracranial metastatic organs						
3–7 vs. 0–2	0.99	0.61–1.62	0.979			
Number of extracranial lesions						
≥ 5 vs. 0–4	1.12	0.67–1.87	0.661			
Liver metastases						
No vs. Yes	0.83	0.46–1.49	0.527			
Primary NSCLC status						
Uncontrolled vs. controlled or absent	1.44	0.88–2.34	0.146			
Local boost at WBRT						
Yes vs. No	1.06	0.59–1.89	0.846			
Dexamethasone equivalent dose over WBRT	1.00	1.00–1.01	0.162			
ICI combined with assigned treatment						
Yes vs. No	0.62	0.33–1.17	0.142			

WBRT, whole-brain radiotherapy; KPS, Karnofsky Performance Status; brain metastases, BMs; NSCLC, non-small cell lung cancer; ICI, immune checkpoint inhibitor.

### Overall survival

For the entire cohort, the median OS after intracranial progression was 8.3 months (95% CI, 6.7–9.3). The median OS for the con-WBRT and an-WBRT groups was 8.4 months (95% CI, 6.5–9.9) and 7.4 months (95% CI, 4.9–13.3), respectively (**Figure 2B**). There was no difference in OS between the two groups (log-rank *P* = 0.38). The results of the univariate and multivariate Cox regression survival analyses are shown in **Table 3**. In the univariate models, mutation type (HR, 2.36; 95% CI, 1.38–4.01), line number of assigned treatment (HR, 1.78; 95% CI, 1.05–3.02), and KPS (HR, 2.40; 95% CI, 1.36–4.23) were associated with OS. Of note, ICI combined with assigned treatment was associated with OS with marginal significance (HR, 0.53; 95% CI, 0.27–1.06; *P* = 0.074). After controlling for the two covariables of treatment assignment and line number of assigned treatment in a multivariable model, EGFR 19/21 or ALK mutation was independently associated with worse OS relative to wild type or unknown (HR, 1.90; 95% CI, 1.06–3.41), and KPS <70 was associated with worse OS relative to KPS ≥70 (HR, 2.34; 95% CI, 1.32–4.17).

**Table 3:** Univariable and multivariable analyses of covariables associated with OS.

	Univariable Analysis			Multivariable Analysis		
Variable	HR	95% CI	*P*	HR	95% CI	*P*
an-WBRT vs. con-WBRT	0.80	0.48–1.33	0.380	0.73	0.42–1.28	0.270
Age	1.00	0.98–1.02	0.946			
Sex						
Male vs. female	0.76	0.46–1.26	0.282			
Histology						
Other vs. adenocarcinoma	0.88	0.40–1.95	0.759			
Smoking status						
Current/former vs. never	0.86	0.52–1.42	0.546			
Stage at diagnosis						
IV vs. I–III	1.21	0.69–2.12	0.510			
Mutation type						
EGFR 19/21 or ALK mutation vs. wild type or unknown	2.36	1.38–4.01	0.002	1.90	1.06–3.41	0.030
Line number of assigned treatment						
Third or later vs. second	1.78	1.05–3.02	0.031	1.55	0.88–2.73	0.127
KPS						
≤ 60 vs. ≥ 70	2.40	1.36–4.23	0.002	2.34	1.32–4.17	0.004
BM status						
Newly diagnosed vs. progressive disease	0.92	0.56–1.52	0.753			
Number of BMs						
> 10 vs. 2–10	1.18	0.71–1.96	0.514			
Size of largest BM	0.89	0.69–1.14	0.349			
BMs with cyst formation						
No vs. Yes	1.24	0.73–2.09	0.425			
Peritumoral edema from BMs						
Moderate or severe vs. absent or mild	0.80	0.48–1.33	0.385			
Extracranial metastases at time of intracranial progression						
Yes vs. No	1.22	0.67–2.23	0.511			
Number of extracranial metastatic organs						
3–7 vs. 0–2	1.46	0.88–2.43	0.142			
Number of extracranial lesions						
≥ 5 vs. 0–4	1.08	0.63–1.82	0.787			
Liver metastases						
No vs. Yes	0.66	0.36–1.23	0.191			
Primary NSCLC status						
Uncontrolled vs. controlled or absent	1.55	0.93–2.59	0.089			
Local boost at WBRT						
Yes vs. No	0.76	0.41–1.39	0.365			
Dexamethasone equivalent dose over WBRT	1.00	1.00–1.00	0.954			
ICI combined with assigned treatment						
Yes vs. No	0.53	0.27–1.06	0.074			

WBRT, whole-brain radiotherapy; KPS, Karnofsky Performance Status; brain metastases, BMs; NSCLC, non-small cell lung cancer; ICI, immune checkpoint inhibitor.

### Adverse events and treatment discontinuation in the anlotinib group

Treatment-related AEs, as determined by the investigator, were reported in 15 patients (44.1%) in the an-WBRT group. **Table 4** lists the AEs (grade 1–2 AEs that affected >10% of the patients and all grade 3–5 AEs). The development of acute or late grade 3–5 AEs was identified in a total of 14.7% of the patients (n = 5), one of which (2.9%) was possibly treatment-related fatal hemoptysis (**Supplementary Table S6**). The full schedules of grade 1 to 2 AEs were illustrated in **Supplementary Tables S7, S8**.

**Table 4:** Treatment-related adverse events in the an-WBRT group.

	an-WBRT(n = 34)				
	Grade, No. (%)				
Adverse event*	1	2	3	4	5
Asthenia	3 (8.8)	3 (8.8)	2 (5.9)	0	0
Anorexia	3 (8.8)	1 (2.9)	2 (5.9)	0	0
Hypertension	1 (2.9)	4 (11.8)	1 (2.9)	0	0
Diarrhea	0	0	1 (2.9)	0	0
Hemoptysis	0	0	0	0	1 (2.9)
Leukoencephalopathy	0	0	1 (2.9)	0	0

*Grade 1–2 adverse events that affected more than 10% of patients in an-WBRT group and all grade 3–5 adverse events are included. Only toxicity of higher grade would be counted in a patient with the same acute and late adverse event but at different levels.

Median time to permanent discontinuation of anlotinib was 5.1 months (95% CI, 2.9–7.4). Kaplan–Meier estimate of time to anlotinib discontinuation was displayed in **Supplementary Figure S1**. Discontinuation of anlotinib because of an AE occurred in six patients (17.6%). AEs leading to discontinuation included leukoencephalopathy, fatal hemoptysis, hematuria, and hematologic toxicity (correspond to patients C and D in **Supplementary Table S6**, patients 21 and 33 in **Supplementary Table S7**), as well as catheter-related thrombosis in the upper limb and infectious pneumonia along with pneumonia-related death (correspond to patients 16 and 14 in **Supplementary Tables S7, S8**). The latter two AEs were considered by the investigator to be not associated with anlotinib. Protocol violation also led to one discontinuation of anlotinib (patient 3). The remaining 24 cases discontinued anlotinib owing to PD. Three patients were still receiving anlotinib treatment at the time of data cutoff.

## Discussion

The preliminary outcomes of this study demonstrated that patients in the an-WBRT group had a longer median iPFS duration of 6.7 months vs. 5.3 months for those in the control group (HR, 0.57, 95% CI, 0.33–0.99), despite no significant difference on OS. A recent study evaluating the therapeutic effect of WBRT in patients with brain-metastatic NSCLC after resistance to EGFR-TKIs reported a median iPFS of 5.4 months. The result of the control group in this current study compared favorably with it (9).

The intracranial efficacy of targeted agents such as EGFR-TKIs or ALK inhibitors has been shown, justifying their use as first-line treatment, in combination with or without cranial RT, in case of brain-metastatic NSCLC with EGFR mutation or ALK rearrangement (10–12). After resistance to these targeted agents, only mildly effective forms of treatment, such as chemotherapy and WBRT if not used previously, are available to control progressive intracranial disease when surgery or SRS is not recommended. For patients without targetable mutations, a dearth of treatment options exists for disperse BMs as well. In the later-line settings, patients refractory to one or more standard systemic therapies could develop highly malignant, rapidly progressive intracranial disease often characterized by the presence of pseudopalisading necrosis and/or peritumoral vasogenic edema via radiology, accompanied by clinical deterioration and poor performance status. These aggregate imaging features indicate, probably, a more critical dependence of these tumors on vascular endothelial growth factor (VEGF) signaling. Moreover, synergism between radiation and the antiangiogenic therapy has been observed in preclinical research; therefore, the efficacy of WBRT, when combined with treatments targeting the pathways involved in tumor and vascular development, may be improved (13). Additionally, WBRT might also enhance penetration of some drugs, thus possibly improving their efficacy (14). The higher efficacy of WBRT plus concurrent anlotinib can lead to improved intracranial disease control, whereas consolidative anlotinib simultaneously controls or stabilizes both extracranial disease and potentially ensuing intracranial micrometastatic disease. The rationales aforementioned could readily explain the prolonged iPFS of the anlotinib group in our study. Thus, the addition of anlotinib to WBRT may represent an appealing intervention against progressive intracranial disease.

The results of this present study suggest that progressive BMs from NSCLC have an intermediate responsiveness to anlotinib in terms of a disproportionate improvement in iPFS relative to OS. It is noteworthy that 11 patients from the con-WBRT group crossed over to receive anlotinib at the time of progression afterward. A consideration is also raised that an actual improvement in OS could be mitigated by a crossover effect. Although OS is not improved, it is argued that delaying intracranial disease progression can lead to delaying physical and psychological BM-associated morbidity and, thus, may confer somewhat clinical benefit.

Corticoids are frequently required to temporarily mitigate peritumoral edema and reduce high levels of intracranial pressure caused by BMs. As there were no intentional protocol amendments on both indications and dosing of the corticosteroid use during WBRT course specially for the anlotinib group at our institution, the trend toward diminished use of dexamethasone may be a consequence of improved tumor control (reduced tumor mass) as well as the decreased permeability of tumor vasculature afforded by anlotinib. Alongside the chance to dramatically mitigate edema from BMs and radiation, anlotinib could effectively abate edema-related symptoms, which is immensely important in particular in managing patients with poor performance status and incapable of self-care. Furthermore, it has been suggested that the use of high-dose steroids might impair the effectiveness of ICIs (15, 16). Reduced dependence on corticoids is of special importance to tackle symptomatic BMs in the immunotherapy era. Antivascular drugs including anlotinib can be considered to treat edema as a new strategy that does not negatively impact the therapeutic efficacy in patients with peritumoral edema while receiving immunotherapy (17).

WBRT combined with concurrent anlotinib followed by anlotinib consolidation may represent an overall safe and cost-effective procedure for NSCLC patients with progressive BMs. In our cohort, the most common toxic effects, such as fatigue and anorexia, were primarily attributed to anlotinib, although WBRT may have also contributed to a certain extent. As these toxic effects notoriously threaten both the quality of life and the level of autonomy in daily activities, it is considered that SRS, when combined with new systemic therapeutics, would be more tolerable than WBRT for selected patients (18). Fatal lung hemorrhage was observed in one patient who scored as having a possibly treatment-related grade 5 toxicity. Although it is difficult to ascribe causality to anlotinib, this drug should be considered with caution for patients with disease having central tumor location, histologic diagnosis of squamous cell carcinoma accompanied by necrosis, and baseline endobronchial tumor involvement (19). The convenience of the simplified chemotherapy-free regimen is also an important consideration, especially in patients with progressive BMs from NSCLC who often have poor performance status, limited mobility, and a short expected life span. Unlike chemotherapy or other therapeutic approaches using intravenously administered agents that often require hospitalization or outpatient treatment with time-consuming trips to the hospital, anlotinib with convenient oral administration and tolerable toxic effects has several advantages, including a greater likelihood of time spent at home allowing patients to maintain a normal family life and family organization during their treatment and cost reduction for home care procedures compared to hospital treatment.

All of the experiences aforementioned support some beneficial effects of anlotinib that cannot otherwise be currently attained for these patients and cannot be overlooked, despite the failure of this agent to improve OS. However, there are limitations to the study. First, this is a single-center retrospective study with a relatively small sample size, which carries with it all of the biases inherent in such an analysis. Second, given the potentially poor prognosis for these patients, it is undeniably challenging to mandate scheduled clinic visits and surveillance imaging. Thus, some data have to be collected via telephone calls and measurement error may occur. Third, it has to be acknowledged that we cannot provide an assessment of toxicities for the control group owing to there being greater difficulties to recollect elaborate toxic effects in patients with an inferior prognosis in a retrospective setting. By contrast, we made a concerted effort to capture the AEs of the anlotinib group in order to facilitate initial safety assessment for this new strategy. Also, toxicities of routine treatment are likely to have been defined in prior studies and need not be intensively collected.

## Conclusions

This present analysis demonstrated that WBRT combined with concurrent anlotinib followed by anlotinib maintenance is associated with better iPFS than that of the contemporary institutional control in advanced NSCLC with intracranial metastasis that progressed or developed after standard systematic treatment. The convenient chemo-free regimen of WBRT plus anlotinib may represent an overall safe and cost-effective procedure in the later-line setting. Randomized prospective studies are needed to further validate these findings.

## Data availability statement

The raw data supporting the conclusions of this article will be made available by the authors, without undue reservation.

## Ethics statement

The studies involving human participants were reviewed and approved by Ethics Committee of Jiangsu Cancer Hospital. The patients/participants provided their written informed consent to participate in this study.

## Author contributions

CK and XZ conceived and designed the study and collected the data. CK interpreted the data, did the statistical design of the study, analyzed the data, generated the tables and figures, and drafted the article. XH, XZ, PQ, SY, MJ, JZ, JX, JFW, and ZG are key members of the multidisciplinary team that evaluated and treated the patients of the study and assisted with data interpretation. XS, JW, and LZ helped to collect the data. All authors had access to all of the data. All authors contributed to the article and approved the submitted version.
