# Supplementary material for: Anlotinib combined with whole-brain radiotherapy in non-small cell lung cancer with multiple brain metastases that progressed or developed after at least one lines of prior treatment
Source: Front Oncol. 2023 Sep 12;13:1169333. doi: 10.3389/fonc.2023.1169333 (PMC10523148; doi:10.3389/fonc.2023.1169333)
Supplement: Supplementary file 1 [file DataSheet_1.zip › Supplementary Tables S1-S8.docx]

**Supplementary Tables S1-S8**

**Supplementary Table 1. Other systemic therapies in combination with WBRT and anlotinib in the an-WBRT group**

| Intervention | No. of Patients | Description |
| --- | --- | --- |
| WBRT+Anlotinib+immunotherapy | 2 | Immunotherapy was given before, concurrently with and after WBRT |
|  | 3 | Immunotherapy was given either before or concurrently with WBRT merely once |
|  | 3 | Immunotherapy was given after WBRT |
| WBRT+Anlotinib+chemotherapy | 4 | Chemotherapy was given 1 cycle before WBRT and additional several cycles after WBRT |
| WBRT+Anlotinib | 22 | No other systemic therapy was given |

**Supplementary Table 2. Systemic therapies in combination with WBRT in the con-WBRT group**

| Intervention | No. of Patients |
| --- | --- |
| WBRT Alone | 9 |
| WBRT+Chemotherapy | 5 |
| WBRT+Chemotherapy+Bevacizumab | 11 |
| WBRT+Target therapy | 9 |
| WBRT+Target therapy+Bevacizumab | 1 |
| WBRT+Immunotherapy | 2 |
| WBRT+Chemotherapy+Immunotherapy | 4 |
| WBRT+Immunotherapy+ Bevacizumab | 1 |

**Supplementary Table 3. Distribution of iPFS events in each group**

| iPFS event classification | con-WBRT (n,%) | an-WBRT (n,%) |
| --- | --- | --- |
| Death | 23 (54.8) | 21 (61.8) |
| Radiologic progression | 16 (38.1) | 7 (20.6) |
| Clinical progression | 0 (0) | 1 (2.9) |
| Censoring | 3 (7.1) | 5 (14.7) |

**Supplementary Table 4. Comparisons of intervals from last brain scan to death between two groups in the subset using death as an iPFS event and having at least one imaging assessment of brain tumor performed after completing WBRT**

| The interval from last scan to death (Months) | con-WBRT (n,%) | an-WBRT (n,%) |
| --- | --- | --- |
| 0 to 3 | 9 (60.0) | 8(57.1) |
| 3 to 6 | 6(40.0) | 6(42.9) |

**Supplementary Table 5. Comparisons of intervals from the end of WBRT to death between two groups in the subset using death as an iPFS event and lack of imaging assessment of brain tumor performed after completing WBRT**

| The interval from the end of WBRT to death (Months) | con-WBRT (n,%) | an-WBRT (n,%) |
| --- | --- | --- |
| 0 to 3 | 4(50.0) | 4(57.1) |
| 3 to 6 | 2(25.0) | 3(42.9) |
| 6 to 9 | 2(25.0) | 0(0) |

|  | Time* | Acute AEs | Late AEs | Description |
| --- | --- | --- | --- | --- |
| Patient A | 12.4 | Asthenia G3 | Asthenia G3 | She tolerated treatment and had not discontinued anlotinib despite the toxic effect. Death was caused by disease progression. |
| Patient B | 13.1 | Hypertension G3  Cough G2  Anorexia G2 | Anorexia G3 | Despite toxicity, he continued anlotinib to retard tumor growth until several days before death. We considered his death was caused by disease progression. |
| Patient C | 5.0 | Anorexia G1  Asthenia G2 | leukoencephalopathy G3  Anorexia G3 | This 57-year-old female began having symptoms including dizziness, nausea, vomiting, and gait disturbance 2 months after completing WBRT. MRI one month later revealed prominent diffuse T2 hyperintensities in the bilateral corona radiate and ventriculomegaly, despite brain metastases being controlled. Anlotinib dose was reduced at 8 mg and continued until the neurocognitive deterioration 4 months after WBRT. The virtual causes of death occurring a further 3 months later could not be veriﬁed. WBRT was considered to be a cause of leukoencephalopathy; however, the effects of anlotinib were not absolutely denied. |
| Patient D | 4.9 | None | Fatal lung hemorrhage G5 | Fatal lung hemorrhage was observed in the absence of local-regional disease progression 4.9 months after anlotinib initiation, and was scored as having a possible G5 toxicity. The patient had a histologic diagnosis of squamous cell carcinoma with necrosis in the left lower lobe and left hilar involvement. After completing anlotinb plus WBRT and radiation of the primary site, he continued maintenance therapy of anlotinib plus chemotherapy. Due to progressive multi-site metastases, he received a dose of camrelizumab as salvage therapy 4 days before fatal hemoptysis. |
| Patient E | 21.5+ | Diarrhea G2 | Diarrhea G3  Asthenia G3 | With a short duration after WBRT, anti-PD-1 immunotherapy was added to anlotinib for liver metastases. Diarrhea led to 3-month discontinuation of anlotinib during the course of treatment. Treatment with anlotinib was ongoing at data-cutoff (indicated by “+”). |

**Supplementary Table 6. Details of grade 3 to 5 treatment-related adverse events**

*Duration of anlotinib exposure (months)

AE=Adverse Event; WBRT=whole brain radiotherapy.

|  | Duration of anlotinib treatment (months) | Acute adverse events |
| --- | --- | --- |
| Patient 3 | 1.2 | None |
| Patient 4 | 3.7 | Hypertension G2 |
| Patient 8 | 3.2 | None |
| Patient 10 | 0.3 | None |
| Patient 12 | 2.7 | None |
| Patient 16 | 0.5 | Catheter-related thrombosis in the upper limb* |
| Patient 19 | 0.4 | None |
| Patient 20 | 2.0 | None |
| Patient 21# | 1.7 | Hematuria G2 |
| Patient 24 | 0.7 | None |
| Patient 25 | 1.0 | None |
| Patient 26 | 3.0 | None |
| Patient 27 | 1.0 | None |
| Patient 33 | 2.9 | Leukocyte count decreased G2  Neutropenia G1  Erythrocyte count decreased G2  Asthenia G2  Cough G1  Anorexia G2  Pain G2 |

**Supplementary Table 7. Details of Grade 1 to 2 acute adverse events in patients with duration of anlotinib administration less than 4 months (n = 14).**

*Considered by investigator to be not associated with anlotinib.

#This case had a history of microscopic hematuria.

|  | Duration of anlotinib treatment (months) | Treatment-related adverse event* |
| --- | --- | --- |
| Patient 1 | 7.6 | Hypertension G2  Asthenia G1  Seizure G2 |
| Patient 6 | 5.9 | None |
| Patient 7 | 19.7+ | Anorexia G1 |
| Patient 11 | 13.6+ | Hypertension G2 |
| Patient 13 | 6.8 | Anorexia G1 |
| Patient 14† | 16.5 | None |
| Patient 15 | 7.4 | None |
| Patient 17 | 7.9 | Gum pain G1  TSH increased G1  Asthenia G1  Hoarseness G1  Hypertension G1  Vomiting G1  Hand-foot skin reaction G1 |
| Patient 18 | 11.0 | Asthenia G2  Anorexia G1 |
| Patient 22 | 5.3 | None |
| Patient 23 | 7.4 | None |
| Patient 28 | 15.1 | Hypertension G2  Asthenia G1  Oral mucositis G1 |
| Patient 30 | 6.6 | None |
| Patient 31 | 6.4 | None |
| Patient 32 | 5.1 | None |

**Supplementary Table 8. Details of Grade 1 to 2 treatment-related adverse events in patients with duration of anlotinib administration more than 5 months (n = 15).**

*Most of these toxicities occurred within 3 months after anlotinib initiation, which can either be managed with symptomatic treatment or persist and become tolerable with or without a dose reduction of anlotinib.

“+” Indicated ongoing treatment with anlotinib at data-cutoff.

†This case died of infectious pneumonia, which was considered non-treatment related.
