# Supplementary figures and images for: Anlotinib combined with whole-brain radiotherapy in non-small cell lung cancer with multiple brain metastases that progressed or developed after at least one lines of prior treatment
Source: Front Oncol. 2023 Sep 12;13:1169333. doi: 10.3389/fonc.2023.1169333 (PMC10523148; doi:10.3389/fonc.2023.1169333)

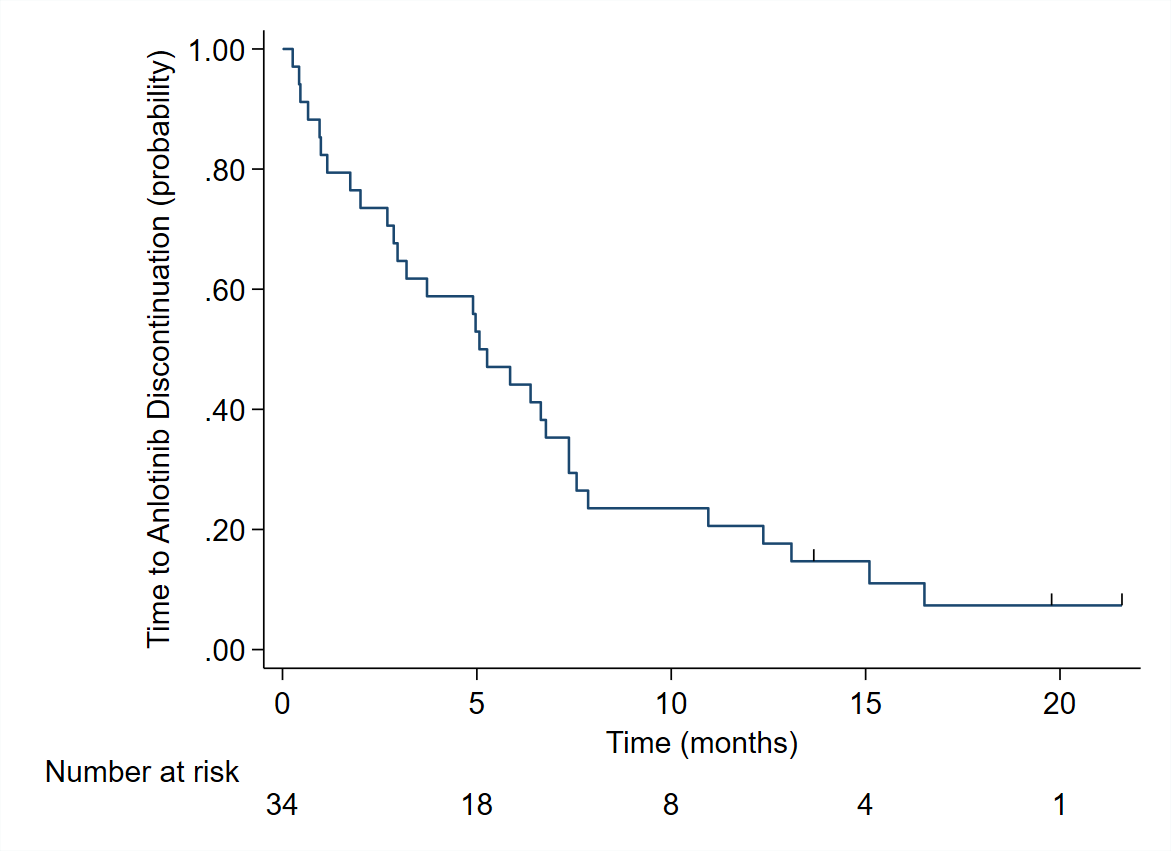


**Supplementary Figure S1. Kaplan-Meier estimate of time to anlotinib discontinuation**

Supplement: Supplementary file 1 [file DataSheet_1.zip › Supplementary Figure S1.docx]
